# Reproducibility of resting-state functional connectivity in healthy aging and brain injury: A mini-multiverse analysis

**DOI:** 10.1162/netn_a_00459

**Published:** 2025-09-22

**Authors:** Hollie A. Mullin, Catherine M. Carpenter, Andrew P. Cwiek, Gloria Lan, Spencer O. Chase, Emily E. Carter, Samantha M. Vervoordt, Amanda Rabinowitz, Umesh Venkatesan, Frank G. Hillary

**Affiliations:** Department of Psychology, The Pennsylvania State University; University of Florida; Moss Rehabilitation Research Institute

**Keywords:** Resting-state fMRI, Functional connectivity, Graph theory, Multiverse analysis, Reliability, Traumatic brain injury (TBI)

## Abstract

Resting-state functional connectivity (RSFC) methods are the most widely applied tools in the network neurosciences, but their reliability remains an active area of study. We use back-to-back 10-min resting-state scans in a healthy aging (*n* = 41) and traumatic brain injury (TBI) sample (*n* = 45) composed of older adults to assess the replicability of RSFC using a “mini” multiverse approach. The goal was to evaluate the reproducibility of commonly used graph metrics and determine if aging and moderate-severe TBI influences RSFC reliability using intraclass correlation coefficients (ICCs). There is clear evidence for reliable results in aging and TBI. Global network metrics such as within-network connectivity and segregation were most reliable whereas other whole-brain connectivity estimates (e.g., clustering coefficient, eigenvector centrality) were least reliable. Analysis of canonical networks revealed the default mode and salience networks as most reliable. There was a notable influence of motion scrubbing on ICCs, with diminished reliability proportional to the number of volumes removed. Choice of brain atlas had a modest effect on findings. Overall, RSFC reproducibility is preserved in older adults and after significant neurological compromise. We also identify a subset of graph metrics and canonical networks with promising reliability.

## Author Summary

In this paper, we examine the reproducibility of resting-state functional connectivity in healthy aging and traumatic brain injury (TBI). We use a mini-verse approach to determine if workflows have a significant effect on resting-state functional connectivity (RSFC) reliability. While the study of RSFC reliability has been previously examined (e.g., in healthy, young adults), it is lacking in the aging and TBI literatures. To our knowledge, this is the first study of back-to-back (consecutive) scans that examine RSFC reliability in healthy aging and TBI. In brief, these data and these analyses do not currently exist in the literature. RSFC reliability remains a vital area of investigation in healthy aging and clinical samples. We believe there are critical contributions that can be made by this paper.

## INTRODUCTION

Resting-state functional connectivity (RSFC) approaches using functional magnetic resonance imaging (fMRI) can identify robust canonical networks in healthy and clinical samples (Buckner et al., 2008; Bullmore & Sporns, 2009). There is recent optimism that RSFC can aid in tracking system level plasticity and developing biomarkers after traumatic brain injury (TBI; Caeyenberghs et al., 2024; Mayer et al., 2011). Although over 100 TBI studies using RSFC point to changes in system-level plasticity after injury, these findings have been challenging to reproduce (Hallquist & Hillary, 2018). For example, both regional hyperconnectivity (e.g., increased network connectivity) and hypoconnectivity (e.g., decrease in network connectivity) have been observed postinjury, and ongoing investigations aim to understand the factors that influence these divergent network responses (Rajtmajer et al., 2022). One source of concern is that the basis for RSFC methods, the blood oxygenation level dependent (BOLD) signal, may be altered after significant brain disruption (Bickart et al., 2023; Caeyenberghs et al., 2024). Moreover, TBI frequently results in axonal injury and gray matter (GM) damage that may disrupt the BOLD signal (Caeyenberghs et al., 2017) and alter the coupling between neural firing and blood flow (Hillary & Biswal, 2007). Similarly, changes in the microvasculature with aging (e.g., vessel hardening, hypertension) may pose challenges for RSFC reliability (Tsvetanov et al., 2021). Given these considerations, the goal in this paper is to determine if RSFC metrics can be reliably observed in older adults with and without neurological disruption.

Establishing the reliability of RSFC also requires methodological standardization, as the myriad approaches to fMRI data processing contribute to nongeneralizable results (Botvinik-Nezer et al., 2020; Poldrack et al., 2017). While there has been significant advancement in the standardization of RSFC approaches (e.g., fMRIPrep), there are still many decision points in network analyses. Two decision points have received significant attention: (a) head motion correction and (b) brain parcellation. Motion correction has been studied extensively, yet there is not a clear consensus on how best to handle it (Bright & Murphy, 2013; Power et al., 2014, 2015). Motion scrubbing based on framewise displacement (FD) is one common procedure that addresses volume-wise motion contamination, but this procedure reduces scan length and relies upon an arbitrary threshold (Phạm et al., 2023). Arguably the most reliable approach to remove motion is through global signal regression (GSR), but it remains a source of controversy (Murphy & Fox, 2017). Motion correction is even more critical in aging and TBI given these samples have an increased propensity for head motion (Power et al., 2014). A second vital decision point for network analysis is brain parcellation. In the study of TBI, the number of nodes used to define brain regions in graph theoretical analyses has ranged from 10 to 67,632 (Hallquist & Hillary, 2018). Additionally, the gross pathology (e.g., hematoma, contusion) associated with TBI can lead to morphological mismatches in brain segmentation and parcellation into standardized atlas space (Bickart et al., 2023; Caeyenberghs et al., 2024; Irimia & Van Horn, 2015). These concerns are similar for aging adults, given that age is associated with significant changes in cortical architecture (Sanders et al., 2022). Taken together, there is a need for evidence demonstrating that RSFC and its associated network metrics are reliable in aging and TBI populations.

To date, the literature establishing RSFC reliability is mostly exclusive to young, healthy adults in their mid to late 20s (Braun et al., 2012; Ma & MacDonald, 2021; Termenon et al., 2016; Tozzi et al., 2020). So, how reliable is RSFC in younger samples? When examining edgewise pairings, there is evidence that the reliability of individual connections is quite low (Noble et al., 2019). In one estimate, single edges had mostly “poor to fair” reliability, 6%–8% were “good,” and less than 1% were “excellent” (Tozzi et al., 2020). While edges can provide important information about brain functioning, it has been observed that these connections tend to miss the higher-level organization of brain networks (Finn et al., 2015). Therefore, other studies have utilized graph metrics to investigate RSFC reliability. Graph theoretical approaches suggest that most canonical networks exhibit “fair to good” reliability, typically when using scans taken days or weeks apart (Braun et al., 2012; Ma & MacDonald, 2021; Termenon et al., 2016). However, these results are affected by processing pipelines, including choice of brain parcellation (Bryce et al., 2021). Although Andellini et al. (2015) provide an overview of graph metric test-retest reliability across several studies investigating healthy adults, individual studies typically limit their analysis to a single or small number of workflows. While the reliability of RSFC in canonical networks is encouraging, it is critical to investigate reliability in aging and postinjury contexts, as both phenomena can substantially alter the organization and functional integration of brain networks (Conwell et al., 2018; Fagerholm et al., 2015).

The current data present an important and unique opportunity to establish the test-retest reliability of RSFC in an aging and TBI sample. We examined test-retest reliability of RSFC by evaluating: (a) graph metrics, based upon (b) separate processing decisions using back-to-back resting-state fMRI (rsfMRI) scans. This is the first evaluation of RSFC reliability using consecutive (same day) resting-state scans in older adults with and without neurological disruption. We leverage a “mini” multiverse approach (for other applications, see Dafflon et al., 2022) for our two major areas of interest: motion correction and brain parcellation. We use 10 graph metrics, eight commonly used brain atlases, and five motion correction pipelines to examine the effect of investigator decisions on RSFC reliability and enhance the generalizability of our findings to other studies. This study was preregistered and a priori goals and hypotheses can be viewed at: https://osf.io/sjaz5.

## METHODS

### Procedure

All study procedures were approved by the institutional review boards of The Pennsylvania State University and Moss Rehabilitation Research Institute. Participants signed an informed consent before participating in the current study. Participants completed a 3-hr research visit. During this visit, participants completed a 60-min fMRI scanning protocol. The protocol included structural scans, resting state scans, and task scans. Participants also completed a battery of neuropsychological tests and self-report questionnaires during the study visit. These methods are reported separately (Vervoordt et al., 2021).

### Participants

The study sample consists of healthy adults and individuals with TBI, all between the ages of 51 and 78 years (*M* = 64.17, standard deviation [*SD*] = 7.61). Participants were recruited from The Pennsylvania State University, Hershey Medical Center, and the Moss Rehabilitation Research Institute. Healthy controls (HCs) were not eligible for this study if they had a history of psychiatric conditions or neurological disorders, including schizophrenia, bipolar disease, substance use disorder, or neurodegenerative diseases. For the TBI group, participants must have a history of complicated mild, moderate, or severe TBI more than a year prior to the study visit. Complicated mild TBI was classified as a Glascow Coma Scale (GCS) score of 13–15, and/or positive acute imaging findings of head trauma. Moderate TBI was classified as a GCS score of 9–12 and/or posttraumatic amnesia (PTA) between 1 and 14 days. Severe TBI was classified as a GCS score ≤ 8 and/or PTA ≥ 15 days. PTA and GCS were determined by participant self-report, interview, and/or medical records.

This sample contained individuals taken from a parent study (Vervoordt et al., 2021). We included only those with back-to-back rsfMRI scans in the current analysis. Based on visual inspection, one participant in the TBI group was removed due to excessive signal loss in their resting-state scans, leaving 45 (32 males) TBI participants and 41 (24 males) HCs in the final sample. Participant characteristics are summarized in Table 1.

**Table 1.** Demographic information

Characteristic	TBI group (*n* = 45)		HC group (*n* = 41)	
	*M*	*SD*	*M*	*SD*
Age	63.40	7.88	65.02	7.10
Education (years)	14.04	2.88	16.37	2.40
Time postinjury (years)	9.02	5.42	–	–
PTA (days)	22.86	27.91	–	–
GCS	11.45	4.11	–	–
% complicated mild	29% (*n* = 13)		–	
% moderate	27% (*n* = 12)		–	
% severe	44% (*n* = 20)		–	
Sex	32 male, 13 female		24 male, 17 female	
Ethnicity	1 Hispanic		0 Hispanic	
Race	34 White/Caucasian, 11 Black/African American		33 White/Caucasian, 8 Black/African American	

Four TBI participants were missing PTA and GCS data but had positive imaging findings of head trauma. These four participants were included in the complicated mild group. Subsequent statistical analyses did not evaluate the effects of TBI severity. PTA = posttraumatic amnesia; GCS = Glasgow Coma Scale.

### Imaging

Imaging data were collected on a Siemens Magnetom Trio 3T Prisma Fit scanner at Penn State Hershey Medical Center (*n* = 8), an identical Siemens 3T Prisma Fit scan at Moss Rehabilitation (*n* = 56), or a Siemens Prisma 3T whole-body scanner at The Pennsylvania State University in the Social, Life, and Engineering Sciences Imaging Center at University Park (*n* = 22). The scanning parameters across all sites were identical.

#### Anatomical scans.

Anatomical structural scans were collected using an Magnetization-Prepared Rapid Gradient Echo (MPRAGE) sequence at a spatial resolution of 1 × 1 × 1 mm voxels with a repetition time (TR) of 2,300 ms, echo time (TE) of 2.98 ms, and flip angle of 9°. Slices were collected interleaved.

#### Resting-state scans.

Participants completed two rsfMRI scans. Each scan was 10 min and the second rsfMRI scan took place immediately after the first rsfMRI scan, resulting in a total of 20 min of data per subject. The length of our resting-state scans was based on standard, 10-min protocols that have provided more reliable network estimates compared with shorter (e.g., 5 mins) scans (Birn et al., 2013). Participants were instructed to keep their eyes open, not fall asleep, and fixate on the white cross in the center of the screen. The resting-state runs included 300 brain volumes each with TR = 2,000 ms, TE = 30 ms, and flip angle = 90. The phase-encoding direction of all resting-state scans were collected anterior to posterior.

#### Preprocessing

Results included in this manuscript come from preprocessing performed using fMRIPrep 23.1.3 (Esteban et al., 2019). fMRIPrep is a standardized, preprocessing pipeline that promotes the adoption of similar preprocessing steps among neuroimaging studies to enhance reproducibility.

Each subject’s T1 scan was referenced throughout the fMRIPrep workflow. The T1w was skull-stripped with a Nipype implementation of the antsBrainExtraction.sh workflow (from Advanced Normalization Tools (ANTs)). Brain tissue segmentation of cerebrospinal fluid (CSF), white matter (WM), and GM was performed on the brain-extracted T1w using FMRIB Software Library (FSL) fast. Volume-based spatial normalization to MNI152NLin6Asym (MNI) space was performed through nonlinear registration with antsRegistration. The transformations from T1w to MNI space were saved and later applied to transform the resting-state scans into MNI space.

For each of the back-to-back resting-state runs found per subject, the following preprocessing was performed. First, a reference volume and its skull-stripped version were generated. Head-motion parameters with respect to the BOLD reference (transformation matrices, and six corresponding rotation and translation parameters) are estimated before any spatiotemporal filtering using FSL mcflirt. The estimated fieldmap was then aligned with rigid-registration to the target EPI (echo-planar imaging) reference run. The field coefficients were mapped on to the reference EPI using the transform. BOLD runs were slice-time corrected to 0.961 s (0.5 of slice acquisition range 0–1.92 s) using 3dTshift from Analysis of Functional NeuroImages (AFNI). The BOLD time-series were resampled into standard space using the stored transformations from T1w to MNI space, generating preprocessed BOLD runs in MNI space to allow for group comparisons.

The eXtensible Connectivity Pipeline-DCAN (XCP-D) was used to postprocess the outputs of fMRIPrep 23.1.3 (Mehta et al., 2023). The first four volumes of the BOLD data were discarded as non-steady-state volumes. Confound regression, using linear regression in Nilearn, was performed using the 36-parameter model from XCP-D. Nuisance regressors included six motion parameters, mean global signal (i.e., GSR), mean WM signal, mean CSF signal with their temporal derivatives, and quadratic expansion of six motion parameters, tissue signals, and their temporal derivatives (Ciric et al., 2018; Satterthwaite et al., 2013). GSR was included in our primary pipeline based on evidence that QC metrics significantly improve, motion confounds are minimized, and the specificity of positive correlations are maximized following GSR (Han et al., 2024; Kassinopoulos & Mitsis, 2022; Murphy & Fox, 2017; Weissenbacher et al., 2009). In addition, GSR removes systematic low-frequency oscillations typically associated with physiological noise (e.g., heart rate and respiration) rather than signal related to neural firing (Liu et al., 2017; Tong et al., 2019). Finally, linear trend and intercept terms were added to the regressors prior to denoising. The BOLD data were despiked with AFNI\u2019s 3dDespike, which corrects for high motion volumes without censoring entire volumes. Despiking was performed prior to nuisance regression and band-pass filtering to minimize the impact of large amplitude changes. Motion scrubbing was not performed in our primary analysis. The time series was then band-pass filtered using a second-order Butterworth filter to retain signals between 0.01 and 0.08 Hz. We used the standard filter in XCP-D recommended by Power et al. (2014) and Satterthwaite et al. (2013). The 0.01- to 0.08-Hz band is commonly chosen because it captures frequencies that are often associated with neuronal signals while filtering out unwanted physiological and scanner-related artifacts (Satterthwaite et al., 2013). Spatial smoothing was not performed during preprocessing, since smoothing is not necessary or recommended for graph theory analyses (Alakörkkö et al., 2017). The preprocessed fMRI data were visually inspected prior to analysis to ensure data quality.

#### Functional connectivity matrices.

**Figure 1.**
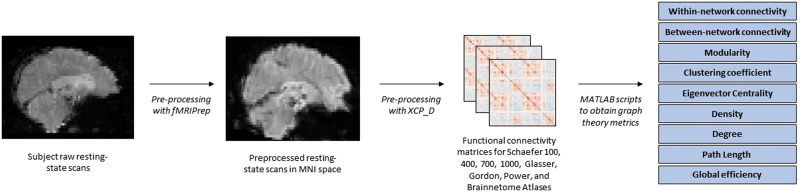
Resting-state processing pipeline. MATLAB scripts refer to graph theoretical scripts from the Brain Connectivity Toolbox.

Weighted functional connectivity matrices, which describe the relationship between brain regions as Pearson correlation coefficients, were created for each subject (see Figure 1). Pearson correlation coefficients are optimal at recovering reliable information regarding network topology (Luppi et al., 2024), which matches how we constructed functional connectivity matrices in the present study. Matrices were created for each subject using the following atlases: Schaefer 100, 400, 700, and 1000 (Schaefer et al., 2018); Glasser (Glasser et al., 2016); Gordon atlas (Gordon et al., 2016); Power (Power et al., 2011); and Brainnetome (Fan et al., 2016). As an example, functional connectivity matrices for the Schaefer 400 atlas would result in a 400 × 400 weighted connectivity matrix per subject, which describes the relationship of the 400 brain regions to one another. The diagonals in the matrices were set to null values (NaNs). In cases of partial coverage, brain regions (nodes) that contained uncovered voxels that were missing signal (values of all zeros or NaNs) were either ignored (when the region had >50.0% coverage) or were set to NaNs (when the region had <50.0% coverage). In other words, brain regions (nodes) with <50.0% voxel coverage were set to NaNs in the connectivity matrix. Negative correlations were transformed to NaNs prior to analysis, given the controversy surrounding the interpretation of negative correlations (Rubinov & Sporns, 2010). Therefore, this study limited its analysis to only positive correlations between brain regions. Pearson *r* correlations in all connectivity matrices were transformed to Fisher *z* values prior to analysis.

### Data Analysis

RSFC was investigated with focus on graph theory metrics recommended by Hallquist and Hillary (2018). These metrics were calculated by adapting scripts from the Brain Connectivity Toolbox (https://sites.google.com/site/bctnet/). The graph theory scripts for this study are publicly available at: https://github.com/holsmon/Masters/tree/main/Graph_Theory_Scripts. All graph metrics are explained in greater detail by Rubinov and Sporns (2010). The 10 graph theory metrics used in the present study are briefly described below:

1. System segregation (measures the relative strength of within-network connectivity compared with between-network connectivity; Chan et al., 2014).
2. Within-network connectivity (strength of a node’s connection to other nodes within the same network).
3. Between-network connectivity (the strength of a node’s connection to other nodes outside of the region’s network).
4. Modularity (measures the density of edges within a network).
5. Characteristic path length (shortest-path distance between a node to all other nodes).
6. Nodal strength (sum of weights of links connected to a node).
7. Degree (number edges per node). Threshold included correlation values > 0.10.
8. Density (fraction of present edges relative to the number of total possible edges for a given node). Threshold included correlation values > 0.10.
9. Clustering coefficient (proportion of connected nodes across all neighboring nodes).
10. Eigenvector centrality (measures the influence of a particular node in a network).

**Figure 2.**
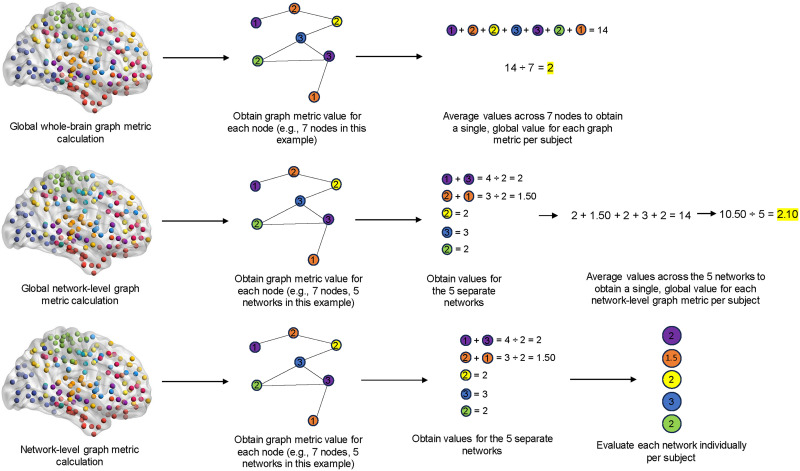
Examples of graph metric calculations used to evaluate reliability. The values and math used here are for demonstration purposes only (mathematical formulas for graph metrics are described in greater detail by Rubinov & Sporns, 2010). Average whole-brain graph metric calculations (2A) refer to characteristic path length, nodal strength, degree, density, clustering coefficient, and eigenvector centrality. Global network-level graph metric calculations (2B) refer to system segregation, within-network connectivity, between-network connectivity, and modularity. Individual network reliability was examined using within-network connectivity (2C). The colors of each node represent the network it is assigned to in this example (e.g., green, purple, orange, blue, and yellow networks).

Figure 2 illustrates examples of the graph metric calculations that were used to evaluate RSFC reliability. Average whole-brain metrics refer to characteristic path length, nodal strength, degree, density, clustering coefficient, eigenvector centrality (see Figure 2a). Average network-level metrics refer to system segregation, within-network connectivity, between-network connectivity, and modularity (see Figure 2b). Individual network reliability was based on within-network connectivity for each of the canonical brain networks for each atlas (see Figure 2c). The Brainnetome (274 nodes) atlas was grouped in 19 networks, and the Schaefer atlases (100, 400, 700, and 1000 nodes) were grouped into 17 networks. The Glasser (360 nodes), Gordon (266 nodes), and Power (231 nodes) atlases were grouped into 12 networks. For more information about these predetermined network assignments, refer to the original papers (Fan et al., 2016; Glasser et al., 2016; Gordon et al., 2016; Power et al., 2011; Schaefer et al., 2018). These network parcels are also available on GitHub (https://github.com/holsmon/Masters/tree/main/Brain%20Parcels).

Test-retest reliability of all graph metrics was determined by intraclass correlation coefficients (ICCs). We utilized ICC (3,1), a two-way mixed-effects model by Shrout and Fleiss (1979). This model is recommended by Koo and Li (2016) to evaluate test-retest reliability, and this model is utilized in other studies that examine functional connectivity reliability (Elliott et al., 2020; Ma & MacDonald, 2021; Noble et al., 2019). ICC values were calculated with RStudio (2023.06.0), and the ICC code is publicly available at https://github.com/holsmon/Masters/tree/main/ICC_Code.

## RESULTS

In the present study, ICCs were interpreted using the following criteria: less than 0.40 “poor” reliability; between 0.40 and 0.60 “fair” reliability; between 0.60 and 0.80 “good” reliability; greater than 0.80 “excellent” reliability (Koo & Li, 2016; Ma & MacDonald, 2021). An ICC was calculated for each graph theory metric to examine its reliability across the back-to-back rsfMRI scans. ICCs were compared with each other using their 95% confidence intervals (CIs). Follow-up *t* tests, regression analysis, and effect sizes (Cohen’s *d*) were conducted by converting ICC values to z scores (Fisher *r*-to-*z* transformation) and analyses were run in RStudio (2023.06.0).

### Graph Metric Reliability

**Figure 3.**
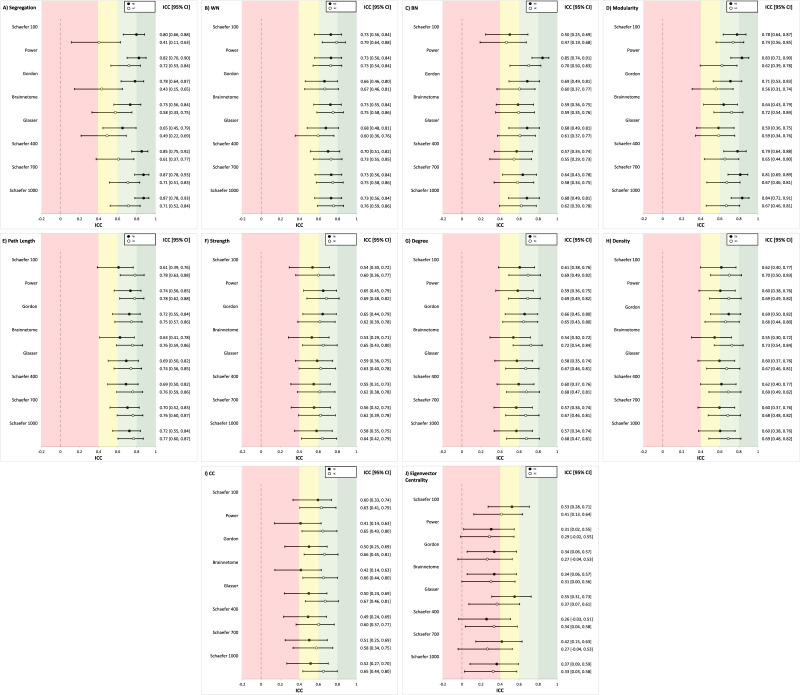
ICCs for each graph metric. Values are ICCs along with 95% CI in brackets. ICCs are provided for the Schaefer, Gordon, Glasser, and Brainnetome atlases. Areas of the figure are color coded using reliability criteria from Ma and MacDonald (2021). Color codes: dark green = excellent; light green = good; yellow = fair; red = poor. WN = within-network connectivity; BN = between-network connectivity; CC = clustering coefficient; EC = eigenvector centrality.

Figure 3 summarizes the ICCs for commonly used graph metrics for the HC and TBI groups across all brain atlases. ICCs for each graph metric were then averaged across the eight atlases to further assess reliability per metric (see Table 2). In Table 2, the range represents the minimum and maximum ICC value for a given graph metric across all atlases. As an example, in the TBI group, segregation has a minimum ICC value of 0.65 for the Glasser atlas and a maximum ICC value of 0.87 for the Schaefer 700 and 1000 atlases (see Figure 3A). In the HC group, segregation has a minimum ICC value of 0.41 in the Schaefer 100 atlas and maximum ICC value of 0.72 in the Power atlas (see Figure 3A).

**Table 2.**
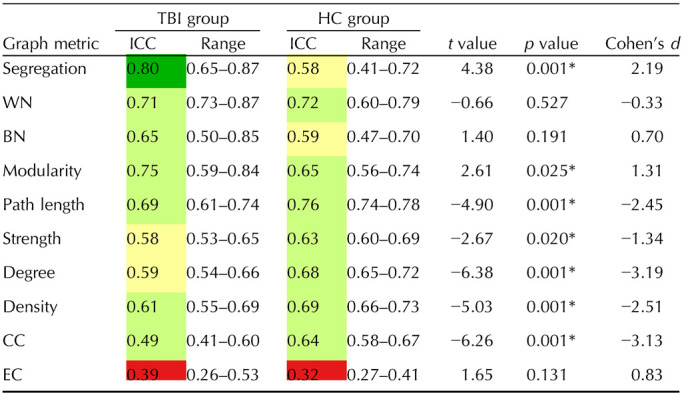
Average graph metric reliability

Mean ICC for each graph metric, based on averaging the ICC values across each graph metric across the eight atlases. Range represents the minimum and maximum ICC value for each graph metric across the eight atlases. The *t* values, *p* values, and Cohen’s *d* were calculated by computing the difference between the TBI and HC groups’ ICCs across the eight atlases for a given graph metric. * indicates statistically significant results. Values are color coded using reliability criteria from Ma and MacDonald (2021). Color codes: dark green = excellent; light green = good; yellow = fair; red = poor. WN = within-network connectivity; BN = between-network connectivity; CC = clustering coefficient; EC = eigenvector centrality.

In order to compare ICCs across the TBI and HC groups, we performed *t* tests on the eight ICCs (per group, across the eight atlases) for each graph metric. System segregation significantly differed between the HC and TBI groups, based on a lack of overlapping CIs. As one example, the TBI group’s ICCs across the Schaefer atlases did not overlap with the HC group’s Schaefer 100 atlas (see Figure 3A). For the TBI group, segregation ICCs were fair to excellent with a mean of 0.80 and a range of 0.65–0.87. In the HC group, ICCs were fair to good with a mean of 0.58 and a range of 0.41–0.72 (see Table 2).

For within-network connectivity, between-network connectivity, modularity, characteristic path length, nodal strength, degree, and density, mean ICCs demonstrated fair to good reliability (ICC = 0.58–0.76) for all atlases across the HC and TBI groups (see Table 2). For all graph metrics (except between-network connectivity), there weren’t significant differences between the groups based on overlapping CIs (see Figures 3B–H); for between-network connectivity (see Figure 3C), there was a significant difference in the ICC between the TBI group’s Power atlas compared with the Schaefer 400 atlas (HC group only) and Schaefer 100 atlas (HC and TBI group). Within-network (TBI ICC = 0.71, HC ICC = 0.72) and characteristic path length (TBI ICC = 0.69, HC ICC = 0.76) were two of the most reliable metrics (ICCs = 0.60–0.87), and their range indicated good reliability (see Figures 3B and 3E). Clustering coefficient and eigenvector centrality had lower ICCs compared with other metrics. For clustering coefficient (TBI ICC = 0.49, HC ICC = 0.64), ICCs ranged from fair to good (ICCs = 0.41–0.67, see Figure 3I). Eigenvector centrality had the worst overall reliability; ICCs fell in the poor to fair range (ICCs = 0.26–0.53) for both groups, and the ICC mean for both groups (TBI ICC = 0.39, HC ICC = 0.32) indicate poor overall reliability (see Figure 3J).

Follow-up *t* tests were conducted to examine if there were statistically significant differences in ICCs between the HC and TBI groups for each graph metric (see Table 2). System segregation reliability was significantly greater in the TBI group compared with the HC group, *t*(14) = 4.38, *p* = 0.001. Similarly, this finding was also significant for modularity, *t*(14) = 2.61, *p* = 0.025. These metrics had effect sizes of 2.19 and 0.70, respectively. In contrast, the HC group had significantly greater reliability for path length, nodal strength, degree, density, and clustering coefficient (see Table 2). Effect sizes for these differences were large, as indicated by Cohen’s *d* values > 0.80. However, unlike system segregation, these graph metrics had overlapping CIs across both groups for all atlases (see Figure 3). No significant differences were found between the groups for within-network connectivity, between-network connectivity, or eigenvector centrality.

### Network Reliability Across Brain Atlases

**Figure 4.**
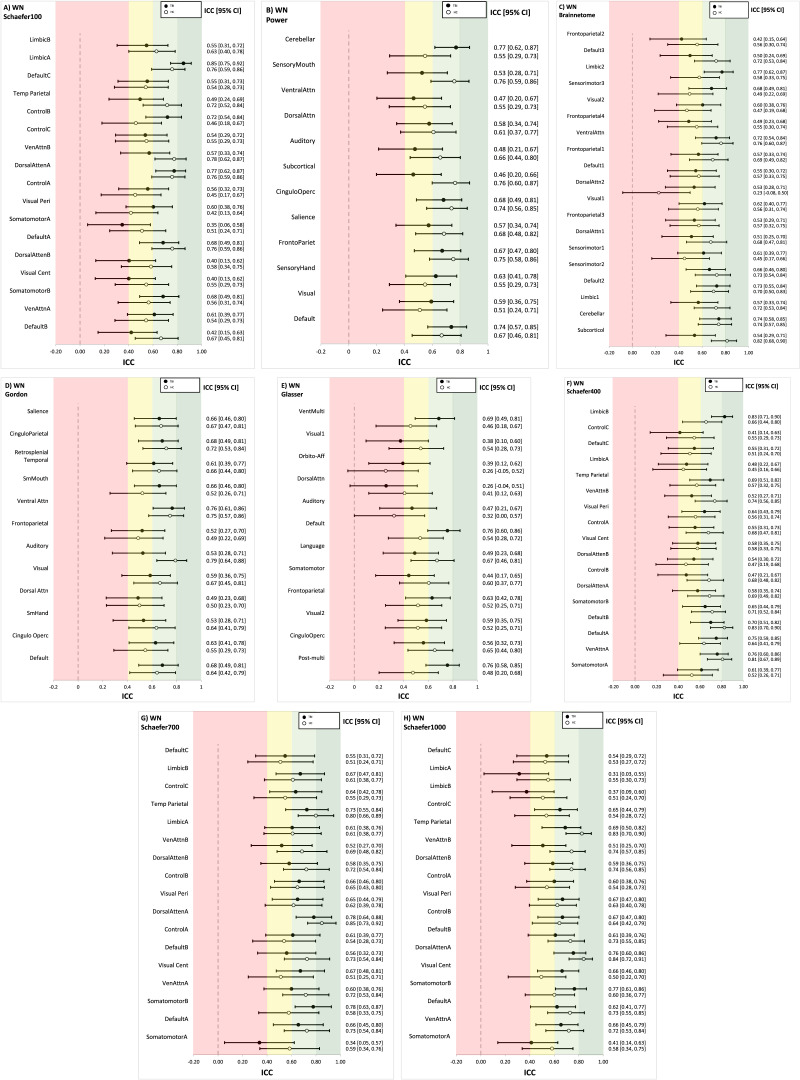
Individual brain network ICCs for within-network connectivity, by atlas. Networks are ordered from small to large (e.g., number of nodes/regions in a given network) for each atlas. Values are ICCs along with 95% CIs in brackets. ICCs are provided for each of the networks across the Schaefer, Gordon, Glasser, and Brainnetome atlases. Areas of the figure are color coded using reliability criteria from Ma and MacDonald (2021). Color codes: dark green = excellent; light green = good; yellow = fair; red = poor. WN = within-network connectivity.

Figure 4 summarizes the ICCs for every brain network for the HC and TBI groups across all brain atlases collapsing across metrics and using within-network connectivity as the common graph metric. Network ICCs were averaged across the atlases to further assess reliability of canonical brain networks (see Table 3). In Table 3, the range represents the minimum and maximum ICC value for a given network based on the included atlases. As an example, in the TBI group, the Auditory network has a minimum ICC value of 0.47 for the Glasser atlas and a maximum ICC value of 0.53 in the Gordon atlas. In the HC group, the Auditory network has a minimum ICC value of 0.32 in the Glasser atlas and maximum ICC value of 0.79 in the Gordon atlas.

**Table 3.**
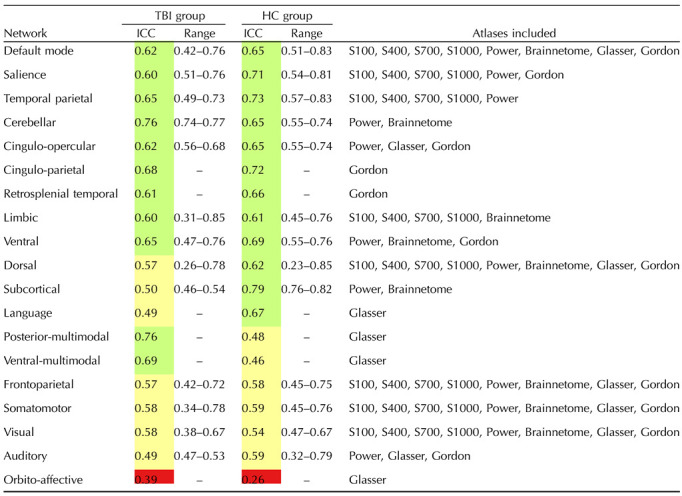
Average within-network reliability

Average ICC for each network, based on averaging the ICCs for each network across atlases. The atlases that were included in calculating the mean for a given network are listed under “Atlases included.” Range represents the minimum and maximum ICC value for each network across the included atlases. Values are color coded using reliability criteria from Ma and MacDonald (2021). Color codes: light green = good; yellow = fair; red = poor. There were no networks with excellent reliability. S100 = Schaefer 100; S400 = Schaefer 400; S700 = Schaefer 700; S1000 = Schafer 1000.

Most networks had ICCs in the fair to good range for both the HC and TBI groups (see Table 3). There were only five networks included in all eight atlases: the visual, somatomotor, frontoparietal, dorsal, and default mode networks (DMN). Among these networks, the DMN had the best reliability with an average ICC indicating good reliability for both groups (TBI ICC = 0.62, HC ICC = 0.65). Other networks that had ICCs in the good range were the salience network (TBI ICC = 0.60, HC ICC = 0.71) which was included in six atlases and the temporal parietal network (TBI ICC = 0.65, HC ICC = 0.73) which was included in five atlases. Although the limbic network had good reliability on average (TBI ICC = 0.60, HC ICC = 0.61) and was included in five atlases, its reliability was more variable with ICCs falling in the poor, fair, good, and excellent ranges (ICCs = 0.31–0.85) across both groups. Larger networks (e.g., networks made up of more nodes), had significantly greater reliability than smaller networks across both groups, *r*(244) = 0.18, *p* = 0.005. This trend was also observed in the TBI group, *r*(121) = 0.19, *p* = 0.033. Although this finding was not statistically significant in the HC group, *r*(121) = 0.17, *p* = 0.063, the same pattern persisted. For each atlas depicted in Figure 4, the networks are ordered by size to illustrate the general trend that larger networks are associated with higher ICCs.

### Motion Correction and Reliability

**Figure 5.**
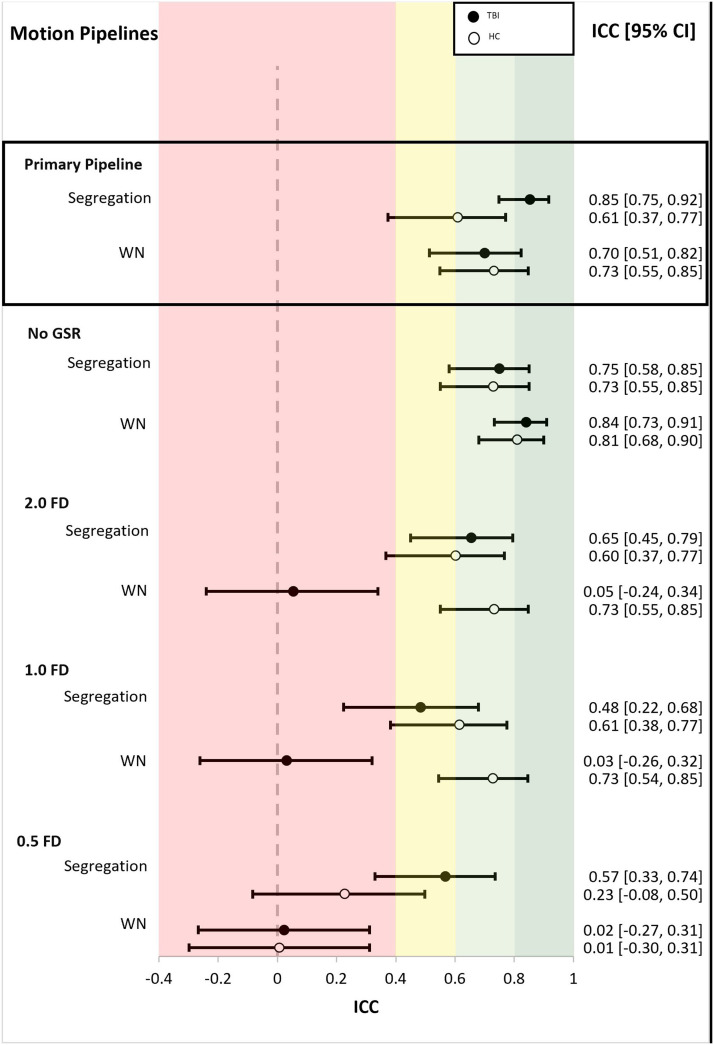
ICCs for motion correction pipelines using the Schaefer 400 atlas. Values are ICCs along with 95% confidence interval in brackets. All pipelines, except “No GSR” used the 36 nuisance regressor pipeline from XCP-D. All pipelines utilized despiking. “Primary Pipeline,” denoted at the top in the black square, refers to our primary pipeline and is included here for direct comparison. “No GSR” used a 32 nuisance regressor pipeline, excluding GSR. 2.0 FD, 1.0 FD, and 0.5 FD refer to the motion scrubbing thresholds (2.0 least strict, 0.5 most strict). Areas of the figure are color coded using reliability criteria from Ma and MacDonald (2021). Color codes: dark green = excellent; light green = good; yellow = fair; red = poor. WN = within-network connectivity.

Figure 5 summarizes the ICCs for 5 motion correction pipelines. The primary pipeline we used to examine graph metric and network reliability was XCP-D’s 36-parameter model with despiking. The primary pipeline was compared with four other motion pipelines, which included one pipeline without GSR and three scrubbing pipelines using thresholds of 2.0, 1.0, and 0.50 FD (see Figure 5). For the sake of simplicity, we compared motion pipeline ICCs using only the Schaefer 400 atlas and two graph metrics that had high reliability in our data: within-network connectivity and segregation.

Results show little influence of GSR on reliability. In a follow-up analysis, we assessed the percentage of total edges with negative Pearson correlation coefficients, both with and without GSR. In the TBI group, we observed 40.36% of edges were negative with GSR and 7.23% were negative without GSR. Similarly, in the HC group, 37.75% of edges were negative with GSR and 4.02% were negative without GSR. Despite these differences, pipelines with and without GSR resulted in reliable ICCs (see Figure 5). However, volume “scrubbing” (i.e., removal of volumes due to suprathreshold motion), had the greatest influence on ICCs, especially for stricter thresholds (see Figure 5). The TBI group had significantly higher motion (FD = 0.32, *SD* = 0.23) compared with the HC group (FD = 0.24, *SD* = 0.11), *t*(126) = 3.01, *p* = 0.003. Therefore, at the 1.0 and 2.0 FD threshold for within-network connectivity, the TBI group had lower reliability than the HCs. There were no significant differences in the amount of motion between the first and second scan for the TBI group, *t*(87) = −0.80, *p* = 0.42, or the HC group, *t*(76) = −1.19, *p* = 0.24. A summary of the average number of volumes scrubbed and the average scan length for the scrubbing thresholds is provided in Table 4. Overall, motion scrubbing resulted in diminished RSFC reliability as a function of the number of volumes removed.

**Table 4.** Motion scrubbing information

Scrubbing threshold	TBI group				HC group			
	Average # volumes scrubbed	*SD*	Average scan length	*SD*	Average # volumes scrubbed	*SD*	Average scan length	*SD*
Scan 1								
2.0 FD	2.18	7.53	9.79	0.25	0.12	0.51	9.86	0.02
1.0 FD	10.69	29.74	9.51	0.99	1.78	4.20	9.81	0.14
0.5 FD	44.78	61.45	8.37	2.05	22.15	30.10	9.13	1.00
Scan 2								
2.0 FD	2.22	6.78	9.79	0.23	0.39	1.02	9.85	0.03
1.0 FD	14.36	32.99	9.39	1.10	3.59	8.71	9.75	0.29
0.5 FD	56.33	72.79	7.99	2.43	29.49	39.52	8.88	1.32

Information regarding the average number of volumes scrubbed based on motion scrubbing thresholds for the back-to-back resting-state scans (Scan 1, Scan 2). Average scan length refers to the average time (in minutes) after scrubbing, and accounts for the first four volumes discarded as non-steady-state volumes. Standard deviation of average scan length is reported in minutes. 2.0 FD, 1.0 FD, and 0.5 FD refer to the motion scrubbing thresholds (2.0 least strict, 0.5 most strict).

### Edge Reliability

**Figure 6.**
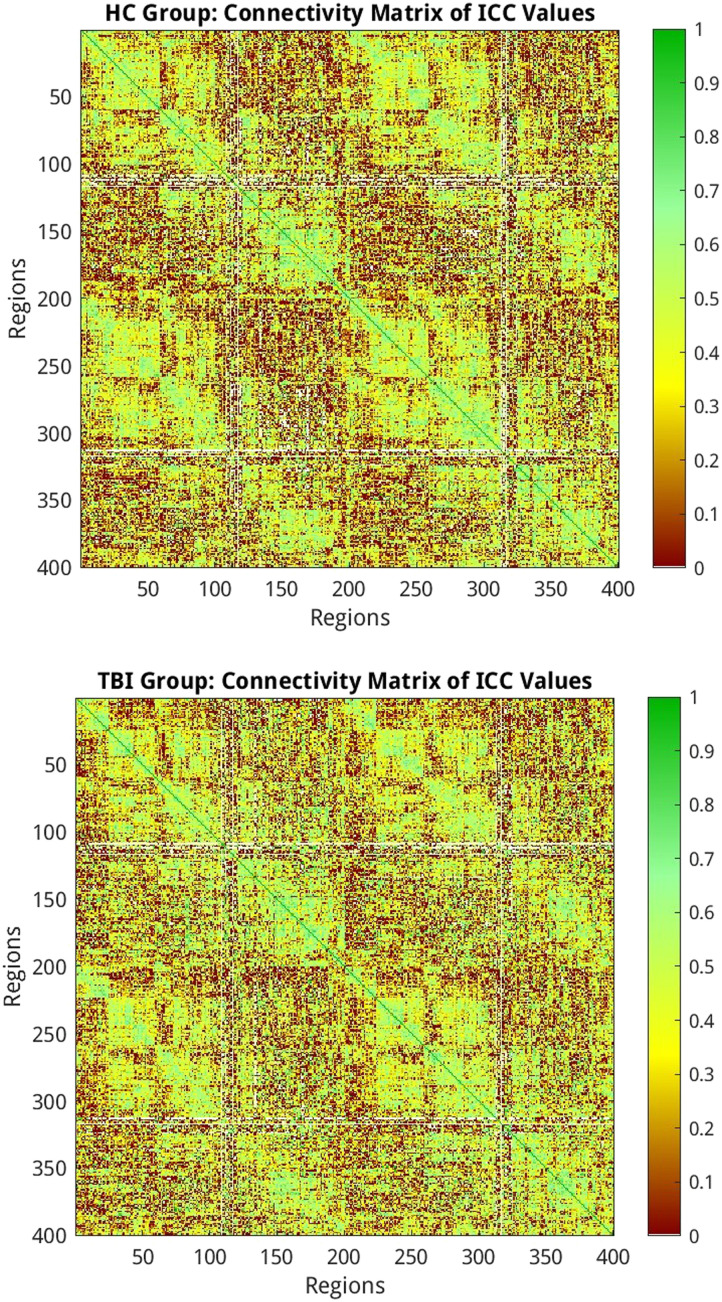
Connectivity matrices representing the reliability of edges in the HC and TBI groups using the Schaefer 400 atlas. The figure is color coded using reliability criteria from Ma and MacDonald (2021). Color spectrum: dark green = excellent; light green = good; yellow = fair; red = poor.

**Figure 7.**
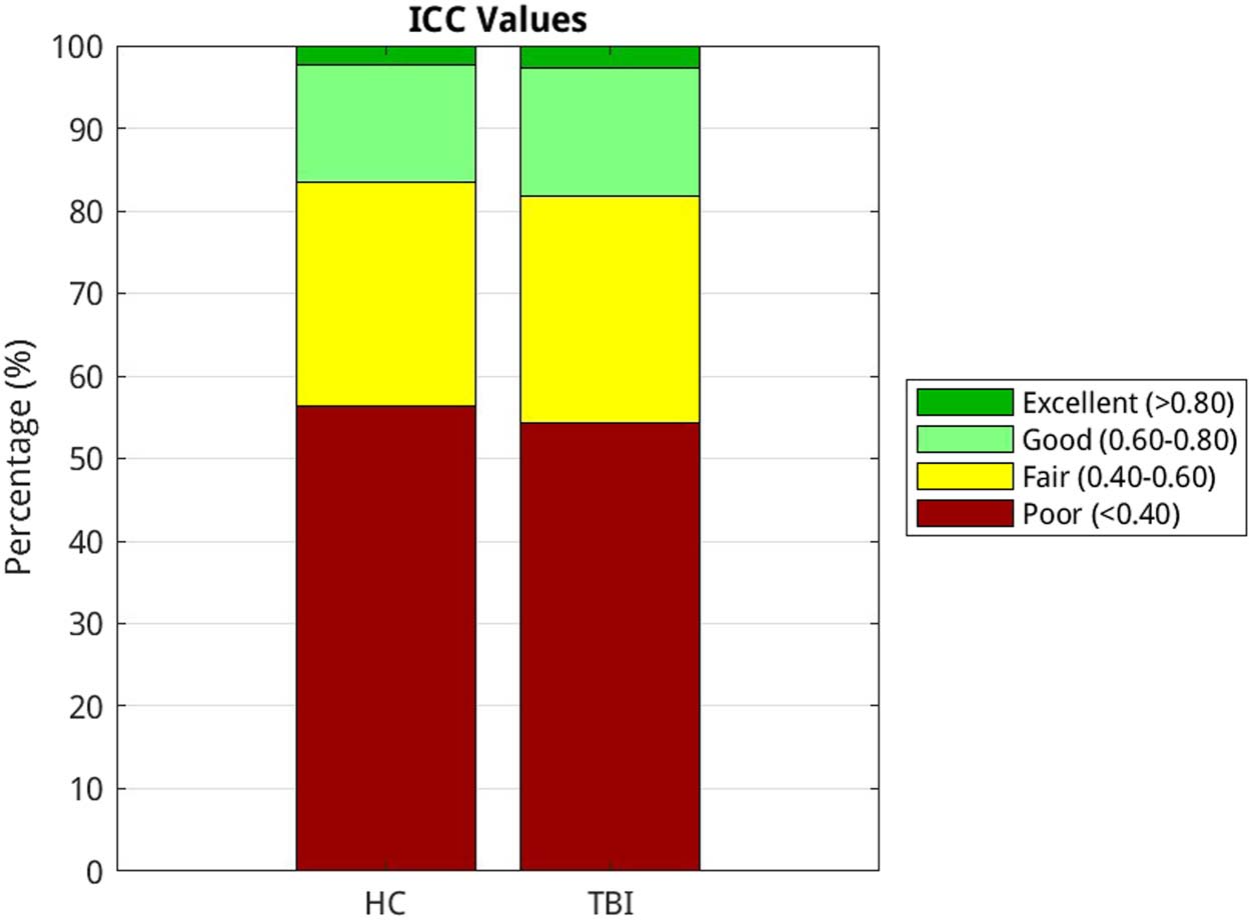
Bar graph representing the percentage of poor, fair, good, and excellent edge reliability in the HC and TBI groups using the Schaefer 400 atlas. Color coded using reliability criteria from Ma and MacDonald (2021). Color codes: dark green = excellent; light green = good; yellow = fair; red = poor.

Although the present study aims to investigate the reliability of graph metrics, we evaluated the reliability of individual edges to establish a benchmark for future RSFC reliability studies in aging and TBI. The custom MATLAB script used to evaluate edge reliability is located here: https://github.com/holsmon/Masters. We examined the reliability of edges in the Schaefer 400 atlas. We observed similar proportions of edge reliability in our TBI and HC samples. In the TBI group, 2.8% of the edges were excellent, 15.5% were good, 27.5% were fair, and 54.3% were poor. In the HC group, 2.3% of the edges were excellent, 14.3% were good, 27.1% were fair, and 56.3% were poor. These results are displayed in Figures 6 and 7.

## DISCUSSION

We used a “mini” multiverse approach to examine the influence of data processing on metric reliability in older adults with and without TBI. The results demonstrate robust findings across several dimensions of analysis, including distinct parcellation approaches and motion correction pipelines. These data also suggest that RSFC can yield reproducible results after significant neurological compromise, something we did not anticipate in our preregistration. We originally posited that significant disruption after TBI could lead to challenges in data processing, greater variability in the BOLD signal, and, ultimately, reduced reliability. To our knowledge, this study is the first to investigate the reliability of RSFC across back-to-back 10-min resting-state scans in a healthy aging and TBI sample. The discovery that RSFC is reliable postinjury is a welcomed finding given the widespread application of RSFC in the clinical neurosciences. Yet, there are instances where RSFC is less reliable. Below, we summarize our findings separately for: (a) graph metrics, (b) brain parcellation (i.e., atlas choice), and (c) motion correction.

### Reliability of Graph Metrics

For standard graph metrics, the most reliable findings were for within-network connectivity, modularity, segregation, and characteristic path length. Clustering coefficient and eigenvector centrality were generally not reliable. These results support prior work that demonstrated modularity shows high reliability, whereas clustering coefficient has been less reproducible (Braun et al., 2012; Termenon et al., 2016).

Unexpectedly, system segregation showed greater reliability in the TBI group compared with the HC group. This finding was observed for modularity as well, though it was less apparent (e.g., overlapping CIs). While the reason for these findings is not entirely clear, it has been observed that enhanced connectivity in TBI (i.e., hyperconnectivity; Hillary & Grafman, 2017) is largely expressed within networks as opposed to between networks (Bernier et al., 2017). Enhanced within-network connectivity is a possible explanation for more reliable graph estimates of segregation and modularity, since nodal strength has been linked to increased reliability (Noble et al., 2019). However, this does not explain why within-network connectivity reliability did not differ between the TBI and HC groups, and we did not observe greater within-network connectivity or strength for the TBI group in the present study (see Supporting Information Table S1). An additional consideration is brain injury results in a loss of network dynamics (Gilbert et al., 2018). Previous studies have found signal variability is important for healthy brain functioning, and variability in RSFC may decline after TBI (Anderson et al., 2023; Raja Beharelle et al., 2012). Therefore, brain connections may actually be *less* flexible postinjury, resulting in more reliable measures of system segregation and modularity (Gordon et al., 2017). While postinjury segregation reliability warrants further research, other studies have also observed high levels of reliability in segregation, making it a notably reliable measure of community-level plasticity (Han et al., 2024).

We also observed greater reliability for path length, nodal strength, degree, density, and clustering coefficient in the HC group compared with the TBI group. TBI is commonly associated with longer average path lengths and lower clustering coefficients (see Supporting Information Table S1; Irimia & Van Horn, 2015) as well as increased variability in these metrics (Nakamura et al., 2009). Increased moment-to-moment variability in network efficiency may contribute to reduced reliability of these graph metrics postinjury. However, it is critical to note that the CIs within each of these graph metrics overlapped. In addition, the mean ICCs for these metrics still fell in the “fair” to “good” ranges, indicating that the difference between the TBI and HC groups may not be practically significant.

The results presented here are consistent with prior findings that suggest graph metrics are more reliable than individual edges (Braun et al., 2012; Noble et al., 2019; Tozzi et al., 2020). We observed similar proportions of edge reliability in both our TBI and HC samples. Therefore, metrics more informed by individual, edge-level inputs may be less reliable. For instance, an individual edge may have less impact on global path length compared with clustering coefficient, which examines triads of edges. Similarly, eigenvector centrality measures the influence of a node in a given network based on the importance of its other edges. Additionally, it is worth considering connections evaluated at the network level (e.g., within-network connectivity, segregation) typically demonstrated higher reliability than edges at the whole-brain level (e.g., degree, density).

### Influence of Brain Atlas on Network Reliability

It is challenging to assess the reliability of canonical networks across atlases given the difference in network representation between atlases and the wide range of results. However, matching our results, other studies have consistently reported that the DMN is one of the most reproducible networks (Elliott et al., 2019; Laumann et al., 2015; Ma & MacDonald, 2021; Noble et al., 2019). In contrast, limbic network reliability was more inconsistent, a finding observed in other studies as well (Elliott et al., 2019; Noble et al., 2019).

We observed some effects of brain parcellation (atlas) on network reliability. Notably, we found that larger networks (i.e., networks containing more nodes) are typically more reliable. For example, the frontoparietal network was reliably observed in the Power atlas (ICCs = 0.67–0.75), but similarly constituted networks were not as reliable using the Brainnetome atlas (ICCs 0.11–0.69). Notably, the frontoparietal network in the Power atlas is made up of 25 regions, whereas the Brainnetome atlas has four frontoparietal networks that contain three, 11, 13, and 14 regions. Another potential reason for atlas-based differences is how the atlas was developed. For instance, the Glasser atlas, which revealed consistently lower ICCs, was originally created using data from 210 young, healthy adults between the ages of 22 and 35 (Glasser et al., 2016). This may not be an ideal match for our sample with a mean age of 64.17 (*SD* = 7.61). In addition, the Glasser atlas was constructed based on cortical architecture, which changes substantially over the lifespan (Sanders et al., 2022). Regardless of the reason, there is clear evidence that differences exist when comparing results between atlases, even when the networks have similar labels. Additionally, the parcellation approach can influence network reliability.

A final point of consideration when evaluating reliability is the underlying function of a given network. The DMN is highly linked to memory retrieval, self-reference, and envisioning the future, all mental activities that are highly likely to be engaged in during a resting-state scan; the DMN is often referred to as the “mind wandering” network (Buckner et al., 2008). Therefore, the DMN may be strongly elicited during a resting-state scan compared with other networks. Our results suggest the DMN could potentially serve as a benchmark for network reliability in RSFC studies. In the case of clinical application, it may serve as a suitable starting point for biomarker development.

### Influence of Motion on Reliability

The use of GSR had minimal impact on reliability. This finding is bolstered by recent work, which found optimal rsfMRI processing pipelines reliably identify network topology both with and without GSR (Luppi et al., 2024). However, motion scrubbing, which removed volumes with high motion based on FD for our dataset, resulted in diminished reliability. While ICCs for both the TBI and HC group were reduced substantially using a strict scrubbing threshold of 0.5 FD, the TBI group was affected to a greater extent using more lenient thresholds; this was driven by higher motion (e.g., greater number of scrubbed volumes) in the TBI group relative to the HCs. This is a critical consideration, given that clinical samples are more prone to head motion in the scanner (Power et al., 2014). Notably, our results match recent studies that indicate head motion scrubbing based on FD negatively impacts reliability and validity of rsfMRI (Kassinopoulos & Mitsis, 2022; Phạm et al., 2023). Additionally, since motion scrubbing reduced the length of the time series, our results are consistent with work that found scans under 10 min have worse reliability compared with longer scans (Elliott et al., 2019). Therefore, there appears to be clear disadvantages to motion scrubbing based on FD with respect to test-retest reliability.

### What Does “Reliability” Indicate?

Finally, a word about what “reliability” indicates in this study is required. Reliability was indexed by ICC values, so any change between the back-to-back scans reduces the ICC, which is interpreted as “less reliable.” However, there are a range of reasons for diminishing reliability. For example, any measurement (e.g., graph metrics) that is sensitive to transient brain states would result in reduced ICC values, even if that phenomenon was captured accurately in the rsfMRI signal. Further complicating the issue, motion and physiological nuisance signals may maintain their own internal consistency, therefore spuriously bolstering reliability (Zeng et al., 2014). Researchers should be aware of these challenges when evaluating the robustness of RSFC.

As a whole, it remains challenging to decipher if differences in reliability are attributable to legitimate, measurable brain dynamics (e.g., intraindividual variability) or spurious instrumentation and/or statistics. Even so, by establishing the reliability of RSFC, these data provide critical foundational information for examining meaningful plastic network response following TBI.

### Limitations

While we anticipate that the current findings hold important implications for reliability of RSFC results, there are limitations to our approach. To make the analyses tractable, we did not explore all preprocessing and postprocessing options (e.g., spatial smoothing, negative correlations). However, recent work has identified that weighted (rather than binary) connectivity matrices constructed with positive correlation coefficients are optimal at recovering reliable information regarding network topology (Luppi et al., 2024), which matches how we constructed matrices in the present study. Therefore, we believe our evaluations of RSFC reliability will be particularly relevant for future research. There are also a range of additional analytical approaches, including other graph metrics and brain atlases, that require validation. Lastly, there are other measures to examine the robustness of functional connectomics that expand beyond test-retest reliability alone, including connectome fingerprinting (Finn et al., 2015; Luppi et al., 2024).

Another factor worth considering is resting-state scan length, which has considerable effects on RSFC reliability (Elliott et al., 2019; Han et al., 2024). In the current study, we observe that 7- and 5-min scans trend toward reduced reliability (see Supporting Information Table S2). Other studies have found that scans less than 5 min have detrimental effects on reliability, which has resulted in 10-min protocols (Birn et al., 2013). However, RSFC reliability does not appear to reach asymptote until scan times are around 20 min in length (Elliott et al., 2019). The current data may underestimate the ceiling of reliability in some graph theory metrics. However, recent work has observed that longer scan time artifactually increases functional connectivity, which could inflate the reliability of RSFC (Korponay et al., 2024). Though we did not observe this phenomenon in the present study (see Supporting Information Table S3), future work should continue to investigate how longer resting-state scans impact RSFC reliability.

## CONCLUSION

The data presented here provides novel information about the reliability of graph metrics and neural network connectivity using back-to-back resting-state scans. Our results suggest largely reliable RSFC results, even in a sample of individuals with significant neurological compromise. However, there were clear examples where reliability diminished based upon data processing strategy or network metric. It is vital to continue exploring factors that may impact RSFC reliability using multiverse approaches. We hope that the data presented in this paper can serve as a reference for future work on RSFC reliability in both the aging and clinical neurosciences.

## SUPPORTING INFORMATION

Supporting information for this article is available at https://doi.org/10.1162/netn_a_00459.

## AUTHOR CONTRIBUTIONS

Hollie Mullin: Conceptualization; Formal analysis; Resources; Software; Validation; Visualization; Writing – original draft; Writing – review & editing. Catherine M. Carpenter: Data curation; Formal analysis; Methodology; Software; Writing – original draft; Writing – review & editing. Andrew P. Cwiek: Data curation; Investigation; Resources; Software; Writing – review & editing. Gloria Lan: Data curation; Formal analysis; Writing – review & editing. Spencer O. Chase: Data curation; Formal analysis; Writing – review & editing. Emily E. Carter: Data curation; Formal analysis; Investigation; Writing – review & editing. Samantha M. Vervoordt: Data curation; Investigation; Writing – review & editing. Amanda Rabinowitz: Investigation; Writing – review & editing. Umesh Venkatesan: Investigation; Writing – review & editing. Frank G. Hillary: Conceptualization; Funding acquisition; Project administration; Supervision; Writing – original draft; Writing – review & editing.

## ETHICS STATEMENT

This study was approved by the Institutional Review Board at the Pennsylvania State University and Moss Rehabilitation Research Institute. Written informed consent was obtained from all study participants.

## FUNDING INFORMATION

Frank G. Hillary, Pennsylvania Department of Health (https://dx.doi.org/10.13039/100004897), Award ID: 4100077082.

## DATA AND CODE AVAILABILITY

Data for this study will be made available upon publication. Analysis and code for the project is available on GitHub: https://github.com/holsmon/Masters.

## Supplementary Material



## TECHNICAL TERMS

Functional connectivity: A type of analysis that examines the connections (Pearson correlation coefficient) between two brain regions.
Resting-state functional magnetic resonance imaging (rsfMRI): Measure of brain activity while a person is at rest and not performing a task.
Graph theory: Study of brain organization using information from functional connectivity matrices. The connection between two brain regions is considered an edge.
Intraclass correlation coefficients (ICCs): Measures the reliability or consistency of measurements within a group, relative to different groups.
Graph metric reliability: The consistency of individual graph metrics across repeated scans using ICCs. In this case, scans are back-to-back.
Connectomics: Study of brain organization, mapping connections between brain regions.
