# Supplementary material for: Reproducibility of resting-state functional connectivity in healthy aging and brain injury: A mini-multiverse analysis
Source: Netw Neurosci. 2025 Sep 22;9(3):1154–75. doi: 10.1162/netn_a_00459 (PMC12548667; doi:10.1162/netn_a_00459)
Supplement: Supplementary file 1 [file netn-9-3-1154-s001.pdf]

# Supplementary Material

**Table S1**

*Graph Metric Differences*

| Graph Metric | TBI Group *Average* | HC Group *Average* | *t-value* | *p-value* | *Cohen's d* |
|---|---|---|---|---|---|
| Segregation | 0.49 | 0.50 | -1.04 | 0.30 | 0.16 |
| WN | 0.40 | 0.44 | -3.88 | <0.001* | 0.60 |
| BN | 0.20 | 0.22 | -3.65 | <0.001* | 0.57 |
| Modularity | 0.12 | 0.11 | 1.37 | 0.17 | 0.21 |
| Path Length | 4.69 | 4.31 | 4.24 | <0.001* | 0.66 |
| Strength | 46.91 | 55.00 | -4.30 | <0.001* | 0.67 |
| Degree | 143.91 | 161.93 | -4.10 | <0.001* | 0.64 |
| Density | 0.35 | 0.39 | -3.93 | <0.001* | 0.62 |
| CC | 0.08 | 0.09 | -3.94 | <0.001* | 0.61 |
| EC | 0.05 | 0.04 | 0.24 | 0.81 | 0.04 |

This table contains information regarding the average graph metric values for the TBI and HC groups. T-tests were utilized to compare the average graph metric values between the groups. * indicates statistically significant results.

**Table S2**

*ICC Reliability for Shorter Scans Schaefer 400 Atlas*

| | TBI Group | | | HC Group | | |
|---|---|---|---|---|---|---|
| | 10 min | 7 min | 5 min | 10 min | 7 min | 5 min |
| Segregation | 0.85 | 0.82 | 0.76 | 0.61 | 0.66 | 0.72 |
| WN | 0.70 | 0.65 | 0.54 | 0.73 | 0.74 | 0.46 |
| BN | 0.57 | 0.56 | 0.41 | 0.55 | 0.71 | 0.29 |
| Modularity | 0.79 | 0.75 | 0.76 | 0.65 | 0.50 | 0.69 |
| Path Length | 0.69 | 0.72 | 0.54 | 0.76 | 0.64 | 0.54 |
| Strength | 0.55 | 0.46 | 0.27 | 0.62 | 0.55 | 0.31 |
| Degree | 0.60 | 0.40 | 0.19 | 0.68 | 0.40 | 0.40 |
| Density | 0.62 | 0.42 | 0.35 | 0.69 | 0.41 | 0.50 |
| CC | 0.49 | 0.44 | 0.05 | 0.60 | 0.46 | 0.20 |
| EC | 0.26 | 0.23 | 0.00 | 0.34 | 0.49 | 0.27 |

This table contains the ICCs for each graph metric in the Schaefer 400 atlas. Our 10-minute (primary) pipeline is included here for reference. We shortened our scan lengths to 7 and 5 minutes (by scrubbing the volumes at the beginning of the scans) to evaluate the effects of shorter scan length on reliability. Values are color-coded using reliability criteria from Ma & MacDonald (2021). Color codes: dark green = excellent; light green = good; yellow = fair; red = poor. WN = within-network connectivity; BN = between-network connectivity; CC = clustering coefficient; EC = eigenvector centrality.

**Table S3**

*Pearson Correlation Coefficient Information*

| Scan # | TBI Group | | HC Group | |
|---|---|---|---|---|
| | *Average* | *SD* | *Average* | *SD* |
| Scan 1 | 0.078 | 0.011 | 0.085 | 0.013 |
| Scan 2 | 0.080 | 0.011 | 0.086 | 0.017 |

This table contains information regarding the average Pearson correlation coefficient for the back-to-back resting-state scans (Scan 1, Scan2). Average refers to the mean Pearson correlation coefficients (whole-brain connectivity matrices) across the subjects using the Schaefer 400 atlas. There were no significant differences in the average Pearson correlation coefficient between the first and second scan for the TBI group $t(88) = -0.69$, $p = 0.49$ or the HC group $t(74) = -0.44$, $p = 0.66$. SD = standard deviation.
